# Role of Polymorphonuclear Leukocytes in the Pathophysiology of Typical Hemolytic Uremic Syndrome

**DOI:** 10.1100/tsw.2007.172

**Published:** 2007-08-10

**Authors:** Ramón A. Exeni, Gabriela C. Fernández, Marina S. Palermo

**Affiliations:** ^1^ Departamento de Nefrología, Hospital Municipal del Niño, San Justo, Provincia de Buenos Aires, Argentina; ^2^ División Inmunología, Instituto de Investigaciones Hematológicas, ILEX, Academia Nacional de Medicina, Buenos Aires, Argentina

**Keywords:** hemolytic uremic syndrome, polymorphonuclear neutrophils, mouse model, inflammation, patients, typical HUS

## Abstract

Thrombotic microangiopathy and acute renal failure are cardinal features of post-diarrheal hemolytic uremic syndrome (HUS). These conditions are related to endothelial and epithelial cell damage induced by Shiga toxin (Stx), through an interaction with its globotriaosylceramide (Gb_3_) receptor. Although, Stx is the main pathogenic factor and necessary for HUS development, clinical and experimental evidence suggest that the inflammatory response is able to potentiate Stx toxicity. Lipopolysaccharides (LPS) and neutrophils (PMN) represent two central components of inflammation during a Gram-negative infection. In this regard, patients with high peripheral PMN counts at presentation have a poor prognosis. In the present review, we discuss the contribution of experimental models and patient's studies in an attempt to elucidate the pathogenic mechanisms of HUS.

## INTRODUCTION

Hemolytic Uremic Syndrome (HUS) is characterized by the triad of thrombocytopenia, microangiopathic hemolytic anemia and acute renal failure, so that it is considered a disease with multiorgan involvement.

Wagner may have been the first to report a case of HUS[1] and Gasser et al. described the features of the disease in 1955[2]. Habib et al. were the first to use the term thrombotic microangiopathy to describe the histopathological lesions in the kidney of a child with HUS[3]. Gianantonio et al. in 1968 described 76 cases of HUS and most important, they instituted the peritoneal dialysis as a treatment in the acute period obtaining a dramatic decrease in the mortality from 50 to 5 %[4]. Kibel et al. in 1968 conducted the first epidemiological study in HUS[5]. Trompeter et al. divided the cases of HUS into those with diarrheal prodrome (D+) and those without diarrhea (D-)[6]. Karmali et al. in 1983 made the main contribution to the understanding of the etiology, pathogenesis and epidemiology of typical HUS, by describing the association between HUS, *Eschericchia coli*, and verotoxin (also referred as Shiga toxin or Stx)[7].

After the description of Karmali, interest was directed to several topics related with Stx role in the pathogenesis of HUS. In 1998 Kaper et al. described the interaction between Stx and the adherence of the bacteria to epithelial cells using intimin dependent and independent mechanisms[8]. Intimin is an outer membrane protein encoded by the eae gen (E. coli attaching and effacing), which is important in the final phase of adherence of the bacterium to the intestinal epithelial cells. There are also several reports on the role of globotriaosylceramide ($Gb_3$) as a cell surface receptor. Binding of Stx to $Gb_3$ is the primary determinant of its cytotoxic effect and results in toxin internalization and cell killing[9].

The lipopolysaccharide (LPS) is a major product of the Gram-negative bacteria that induces the clinical syndrome of septic shock and renal cortical necrosis. Both Stx and LPS may be absorbed from the inflamed gastrointestinal tract and induce synergistic effects in Stx-induced HUS. Louise et al. suggested that Stx may enhance the procoagulant effect of LPS [10]. Renal thrombotic microangiopathic (TMA) lesions, mimicking those seen in human HUS, have been reproduced in rabbits and mice infused with LPS. Many substances, including prostaglandins, cytokines, vasoactive and procoagulant factors are stimulated by LPS[11]. Stx-induced endothelial injury is the primary pathogenic event and LPS, chemokines and cytokines released by inflammatory cells (Interleukin (IL)-6, IL-8)[12] or injured cells (basic fibroblastic growth factor)[13] may contribute with this process.

Although Stx is the main pathogenic factor and necessary for HUS development, several authors have reported the importance of the inflammatory response in the development of HUS. In this regard, LPS and polymorphonuclear leukocytes (PMN) represent two central components of inflammation during a Gram-negative infection. In this review the importance of PMN in the pathophysiology of the typical form of HUS will be considered, discussing evidence arising from experimental models and from studies in patients with HUS.

## CONTRIBUTION OF PMN TO HUS DEVELOPMENT

Children with HUS usually show a high peripheral blood PMN count at presentation, a fact that is not surprising since PMN are a prominent component of the acute inflammatory response triggered as a consequence of an infectious process[14,15]. However, the contribution of PMN to the disease has been suggested, since a high peripheral blood PMN count at presentation is a positive predictor of a poor outcome[15,16,17,18,19], and PMN influx into the renal cortex of HUS patients has been related to more severe cases and death[20]. In addition, activation and degranulation of PMN have been indirectly demonstrated by the presence of high levels of elastase and IL-8 in the serum of patients. Elastase is the major lysosomal proteinase liberated by activated PMN[16,21,22]. On the other hand, IL-8 is a cytokine produced by activated PMN that promotes their adhesion and migration *in vivo,* and delays PMN apoptosis[23,24,25,26]. Moreover, PMN from HUS patients have increased adhesive capacity *in vitro* and show reduction in their granule content as demonstrated by ultrastructural studies[18,27].

PMN are essential for host defense against microbial infections but can also be associated with pathologic side effects of tissue destruction, and especially with endothelial cell damage[28,29]. Because endothelial dysfunction appears to be an important factor in the sequence of events leading to the microangiopathic process of HUS, and since PMN possess mechanisms that can mediate tissue injury, it is reasonable to assume that activated PMN may be involved in the pathogenesis of HUS and contribute to endothelial damage in HUS.

Different animal models have been used to study the pathogenic mechanism of HUS[30,31,32,33]. The lack of an animal model that reflects all features of human HUS is a true limitation, and it is possibly related, at least in part, to interspecies differences in the expression of the Stx specific receptor, $Gb_3$. In spite of the fact that the $Gb_3$ receptor is absent in the glomeruli of mice, the mouse model of HUS by systemic injection of Stx2 reproduces the acute renal lesions, mainly by inducing tubular necrosis. Glomerular alterations are also observed, although they are due probably to systemic alterations such as the widespread thrombosis and the decreased renal flow secondary to hemodynamic imbalance[34,35]. Moreover, the mouse model reproduces other systemic alterations characteristic of HUS patients, such as platelet activation, thrombocytopenia and neutrophilia[35,36]. Therefore, and in spite of its limitations, the murine model of HUS, by intravenous injection of purified Stx2, is a useful tool that allows the investigation of early events in the course of HUS. These events are very difficult to study in patients because most of them have probably already occurred in children before they are diagnosed. Moreover, the mouse model of HUS has made it also possible to investigate the role of the inflammatory response in the development of the disease.

The contribution of the mouse model to elucidate the underlying mechanisms leading to circulating leukocytosis as well as the role of PMN in the pathogenic mechanisms of HUS is discussed below.

## EVIDENCES OF PMN CONTRIBUTION (TO HUS DEVELOPMENT) FROM THE MOUSE MODEL

A sustained neutrophilia has been reported after one intravenous injection of pure Stx2 in mice, without alterations in other leukocyte populations[36]. The mechanisms underlying Stx2-induced neutrophilia include an increase in the proliferation of myeloid progenitors, an acceleration of leukocyte appearance into the peripheral circulation and a reduced migration into tissues[37]. As indicated by differential count of peripheral myeloid subpopulations, Stx2 had an effect only on the neutrophilic population, which egressed not only at a faster rate but also at an earlier stage of maturation[37]. The impaired migratory capacity of PMN may be attributed to alterations in the environmental signals rather than to PMN deficiency, since *in vitro* PMN migration towards chemoattractans was enhanced[38]. Furthermore, fluorescent PMN from Stx2-treated mice showed an increased migration to peritoneum, induced by thioglycolate, when injected into normal mice[37].

In addition, a positive correlation between neutrophil percentage and renal damage assayed as plasmatic urea was found, suggesting a role for PMN in the pathogenesis of HUS [36]. Most important, it has been recently reported that mice depleted of PMN, by treatment with a specific granulocytic anti-serum, presented a reduced sensitivity to Stx2-dependent renal toxicity and lethal effects[37]. Then, it can be concluded that neutrophilia is not an epiphenomenon, but participates directly in the pathogenic mechanism of Stx2.

*Ex vivo* analysis of peripheral PMN from Stx2-treated mice after 72 hours, showed a clear pattern of functional and phenotypic activation. In fact, these PMN showed increased membrane expression of the adhesion molecule CD11b/CD18, as well as an increase in PMN adhesion both, *in vivo* to the endothelium and *in vitro* to extracellular matrix proteins (fibrinogen, bovine serum albumin, collagen)[36,38]. In addition, they presented enhancement of their cytotoxic capacity, evidenced by an increase in antibody-dependent cellular cytotoxicity (ADCC) and production of reactive oxygen species (ROS)[36,38]. Furthermore, the apoptotic rate of PMN from Stx2-treated mice was increased when compared to controls[38]. All these observations suggest that mechanisms coupled to PMN activation are exacerbating Stx2 toxicity in the mouse model. Moreover, as HUS is a systemic disorder, it cannot be excluded that PMN exacerbate systemic Stx2-induced endothelial cell damage, causing a more pronounced generalized thrombotic state that, in turn, can also contribute to renal injury.

On the other hand, Stx2 injection also generates an anti-inflammatory reaction, secondary to the early inflammatory response, mainly characterized by endogenous glucocorticoid (GC) secretion[39]. It has been demonstrated that endogenous or exogenous GC can attenuate Stx2 toxicity and HUS severity in mice[39]. Although several mechanisms may be involved in GC protection against Stx toxicity, it has been demonstrated that GC-protection is mediated, at least in part, by restraining PMN activation. In fact, the blockade of endogenous GC action through the administration of a GC-receptor antagonist simultaneously with Stx administration, induced in PMN a higher generation of ROS after phorbol-myristate-acetate (PMA) *in vitro* stimulation, a higher adhesion to BSA and collagen type I (CTI), and a higher migration towards N-formyl-methionyl-leucyl-phenylalanine (fMLP), compared to PMN from mice treated with Stx2 alone. It was concluded that Stx2 activates PMN and that the absence of endogenous GC-action enhances this activation, suggesting that endogenous GC can at least partially, counteract PMN inflammatory functions.

Stx-producing *E. coli* (STEC) colonize human colon epithelium and induce acute colonic inflammation, but do not invade the epithelial cells. Since acute inflammatory infiltration of the gut and the presence of leukocytes in feaces are seen in many STEC-infected patients, several studies were conducted to analyze the role of PMN in the intestine. Although Stx does not bind to normal or inflamed human colon epithelium *in vivo*, several pathogenic factors of STEC have been demonstrated to induce or up-regulate the expression of epithelial cell pro-inflammatory chemokines, which was accompanied by a sub-epithelial influx of PMN[40,41]. Moreover, Hurley et al. demonstrated that STEC interaction with a cellular line of intestinal epithelia (T84), induces the basolateral-to-apical transmigration of PMN, which in turn, significantly increased the movement of Stx1 and Stx2 across polarized T84 cells in the opposite direction[42]. The amount of Stx crossing the T84 barrier was proportional to the degree of PMN transmigration, and the increase in Stx translocation appears to be due to increases in paracellular permeability caused by migrating PMN. Additionally, PMN recruitment in the intestine may also increase the risk of HUS, because they were found to induce the Stx2 prophage *in vivo* and to augment Stx2 production, mainly through the production of $H_2O_2$[43].

## EVIDENCE OF EARLY ACTIVATION IN PMN FROM HUS PATIENTS

Two main mechanisms mediated by activated PMN are involved in host defense and tissue damage. One is the production of toxic ROS by the NADPH oxidase system, also referred as the respiratory burst, which is initiated by phagocytosis of opsonized organisms, cell adhesion, aggregation and as a response to chemotactic substances. The other one comprises the proteolytic capacity of enzymes stored in granules. The activation of PMN results in the mobilization of these intra-cytoplasmatic vesicles, resulting not only in the release of granule content but also in the up-regulation of adhesion proteins in the plasma membrane[44]. This rapid up-regulation of membrane molecules can be followed by their down-regulation or shedding, usually mediated by proteases released during degranulation[45,46].

Studies on circulating PMN from HUS patients in the acute period evidenced that the phenotype and functionality of PMN is altered in these patients. They have a reduced expression of membrane molecules (CD16 and CD11b) involved in adherence and inflammatory responses, and a reduced intracellular content of molecules and enzymes found in specific (CD66b) and azurophil (myeloperoxidase and β-glucuronidase) granules[47,48]. PMN also show impaired degranulation capacity upon cytokine stimulation, and decreased cytotoxic responses, such as ADCC and ROS production after direct PKC stimulation. These data suggest that PMN are partially deactivated, probably due to a post-activation exhaustion process[47,48]. These derangements are unlikely mediated by circulating soluble factors that down-modulate PMN's functions, since incubation of healthy adult donor PMN with plasma from HUS children did not render them hypo-responsive [47]. As the reduction of CD16 and impaired functionality of PMN have been associated with cell death, the percentage of spontaneous apoptosis of these cells has been investigated. The entire population of PMN in HUS patients shows an increased survival rate compared to healthy control children. Moreover, patients who presented more deactivated PMN, are those that showed the lowest percentages of apoptosis[49], indicating that deactivated PMN are not dying cells. Taking into account these results, it has been hypothesized that circulating PMN from HUS patients on admission are low responders as a consequence of a previous activating process, which triggered the respiratory burst and the release of proteases associated to degranulation. This process lead PMN to a deactivated or "exhausted" state, similar to what is found for platelets, which circulate in a degranulated form as a consequence of a strong thrombotic stimulus prior to the moment of hospitalization[50,51,52,53,54].

## MECHANISM OF PMN ACTIVATION DURING HUS

The discussion of whether PMN activation during HUS, both in humans and animal models, is a consequence of the direct effect of Stx-binding, or is mediated indirectly through the endothelial activation secondary to Stx-direct damage, has been clarified by recent work.

Some authors[55,56] have described Stx bound to PMN from HUS patients, several days after it was no longer detectable in stools. In addition, they and others also reported that Stx was able to bind to PMN *in vitro*, even when they lack of $Gb_3$ or other known specific Stx receptor[57,58]. However, Geelen et al.[59] have recently reported the lack of specificity in the binding of Stx to PMN, both *in vitro* or in HUS patients, and ascribed this non-specific binding to possible changes in PMN membrane due to activation. Moreover, the hypothesis that Stx has direct effects on PMN viability and function has been examined *in vitro* by measuring apoptosis, necrosis, phagocytosis, and spontaneous and PMA-stimulated production of ROS upon Stx incubation. Although some authors reported that Stx can influence the respiratory burst or the apoptotic rate of PMN *in vitro*[60,61], there is growing evidence demonstrating that neither Stx1 nor 2 have effects on the PMN priming or PMN degranulation, even at high concentrations or after pre-incubation with activating stimuli[47,59,62,63].

All the data exposed above suggest that it is likely that activation of PMN in HUS is mediated indirectly by injured or activated endothelial cells and other inflammatory cells rather than by direct interaction with Stx. In this regard, Stx is able to bind to and trigger cellular responses in endothelial cells (amplified in the presence of inflammatory factors)[64,65], renal epithelial cell[66,67], and monocytes[68]. In this last population, Stx binding triggers the secretion of several chemokines and cytokines (tumor necrosis factor-alpha (TNF-α), IL-1β, IL-8, RANTES, tissue factor) which in turn increase endothelial susceptibility, and activate platelets and neutrophils. The injury or disruption of the endothelial lining of blood vessels leads to profound alterations in the haemostatic state and inflammation. In fact, the exposure of the subendothelium, which contains von Willebrand factor, collagen, and fibrinogen induces platelet aggregation and activation[69]. Activated platelets interact with leukocytes, both neutrophils[70] and monocytes[71], and release certain chemokines, present in platelet granules, that also potentiate the inflammatory process[72]. These include platelet factor 4 (PF-4), macrophage inflammatory protein (MIP)-α, RANTES, β-thromboglobulin, monocyte chemoattractant protein (MCP)-3, and IL-8. On the other hand, leukocytes tether to and roll on altered endothelium, producing a link between inflammatory and thrombotic processes.

This complex interplay among inflammatory and thrombotic cascades initiated by Stx direct binding to monocytes and endothelial cells, may be enough to explain neutrophil (as well as platelet) activation (Figure 1).

## EVIDENCE OF PMN INVOLVEMENT IN PATHOGENIC MECHANISM OF HUS

During an acute inflammatory response, PMN bound to the endothelial surface may be pathological, since adherent and activated PMN may cause vascular occlusion and damage. Van Setten et al. found only a minor infiltration of PMN within the renal tissue of patients[73], and Inward et al. reported an increased number of PMN within the glomeruli of patients, only in fatal cases of HUS[20]. In agreement with these observations adherent or infiltrated PMN were not observed within the kidney of Stx2-treated mice[37]. However, the absence of PMN does not discard the potential damaging role of reactive oxygen species or proteases released upon PMN activation. Moreover, the importance of PMN in the course of HUS is also supported by the finding that the functional state of PMN in the acute period of HUS patients is inversely correlated with the severity of renal damage[49]. Therefore, PMN from patients with the highest degree of renal insufficiency showed the lowest levels of CD11b, CD66b, intracellular myeloperoxidase and ROS production[49]. The inverse correlation between PMN functionality and severity suggests that a strong initial activating stimulus, that induces a strong activation of PMN followed by a subsequent

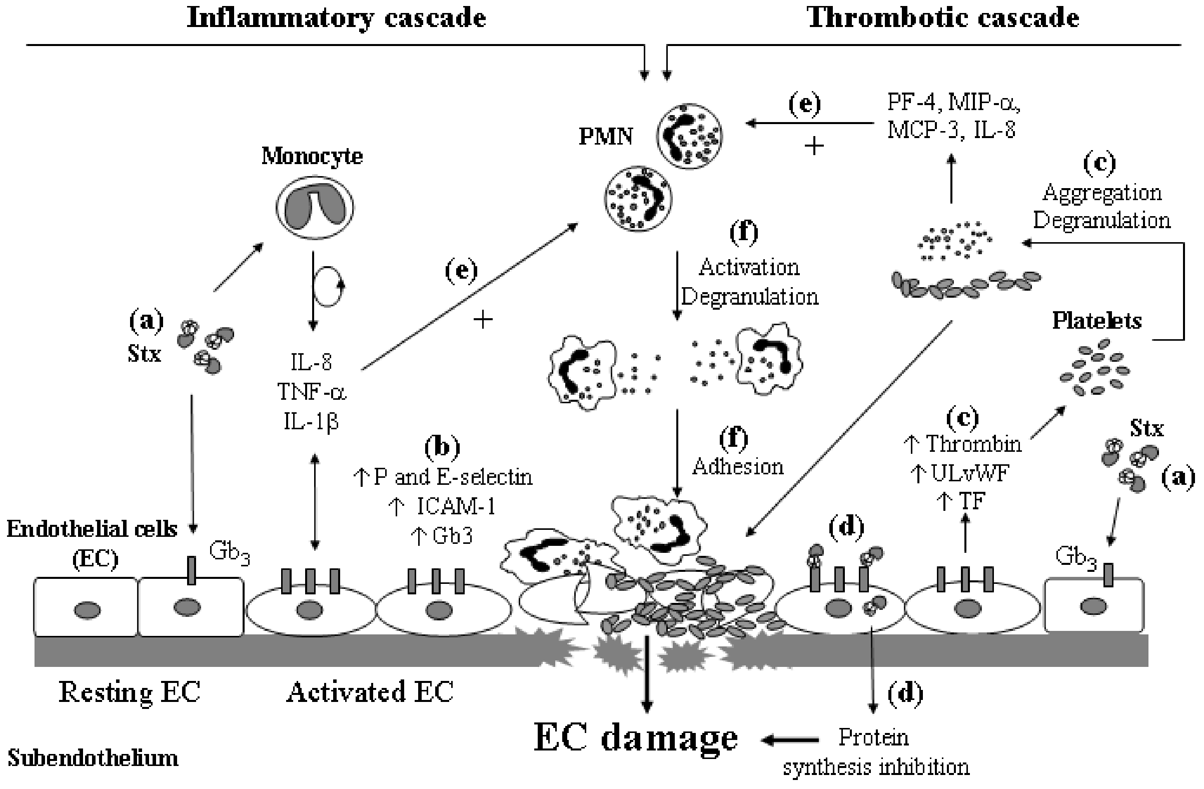

**FIGURE 1.** Scheme depicting the sequence of events leading to PMN activation and endothelial damage in HUS. (a) Stx binds to monocytes and endothelial cells (EC) promoting their activation and secretion of cytokines and chemokines. (b) Cytokines released cause up-regulation of adhesion molecules and of the $Gb_3$ receptor in EC. (c) Activation of EC leads to secretion of thrombotic factors that induce platelets aggregation and degranulation. (d) Stx internalization in activated EC induces inhibition of protein synthesis. (e) Factors released by activated endothelium, monocytes and platelets collaborate in PMN activation. (f) Activated PMN release their granule content, produce reactive oxygen species, adhere to EC and, together with platelets, potentiate Stx-induced EC damage. ULvWF: ultra large von Willebrand factor, TF: tissue factor, TNF-α: tumor necrosis factor- α, IL-1β: interleukin-1 β, PF-4: platelet factor 4, MIP- α macrophage inflammatory protein- α, MCP-3: monocyte chemoattractant protein-3, IL-8: interleukin-8, ICAM-1: intercellular adhesion molecule-1.

impairment, will derive in a more severe clinical course (Figure 2). The intensity of PMN activation, proportional to the level of PMN deactivation, may probably result from different interrelated factors, such as initial bacterial inoculum, Stx producing capacity of the bacteria ingested, specific characteristic and degree of activation of the thrombotic and inflammatory responses due to individual variability.

## CONCLUSIONS

HUS is a complex disease with multiple pathogenic scenarios that interconnect with each other resulting in a characteristic clinical outcome. PMN and their potential cytotoxic capacity are central in the pathophysiology of HUS by exacerbating Stx2 toxicity in the early stages of the disease. Although it is reasonable to consider the targeted blockade of factors contributing to HUS pathogenesis as therapeutic strategies for STEC treatment, by the moment the child seeks medical attention, PMN already show a reduced toxic potential, so that the success of therapies based on the reduction of the inflammatory response will depend on a prompt diagnosis and treatment.

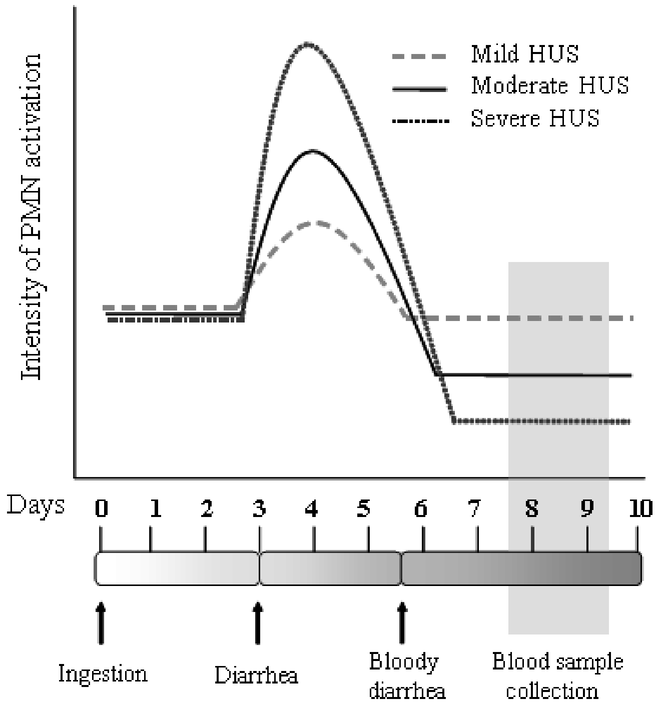

**FIGURE 2.** Intensity of PMN activation during the course of Shiga toxin-producing *E. coli* infection. Approximately, liquid diarrhea starts 3 days after ingestion of the Shiga toxin-producing bacteria, indicating the colonization of bacteria. Stx is synthesized and secreted by the bacteria attached to the intestinal mucosa, and the damage produced leads to bloody diarrhea. Usually children are hospitalized 3 or 4 days after the beginning of bloody diarrhea. PMN activation probably occurs before hospitalization, and at the moment of sample collection PMN are found deactivated in degrees that depend on the intensity of their previous activation. PMN form patients with mild renal damage (mild HUS) will show the lowest activation, patients with moderate renal dysfunction (moderate HUS) an intermediate activation and severe cases of HUS (severe HUS) the highest degree of PMN activation. This will derive in minor, intermediate and marked alterations in PMN phenotype and functionality for mild, moderate and severe cases of HUS, respectively, at the moment of sample collection.
